# Case Report: Factor VII Deficiency Presented With Cephalohematoma After Birth

**DOI:** 10.3389/fped.2021.755121

**Published:** 2021-10-15

**Authors:** Yuan-Chun Lo, Ching-Tien Peng, Yin-Ting Chen

**Affiliations:** ^1^College of Medicine, China Medical University, Taichung, Taiwan; ^2^Division of Pediatric Hematology and Oncology, China Medical University Children's Hospital, China Medical University, Taichung, Taiwan; ^3^Division of Neonatology, China Medical University Children's Hospital, China Medical University, Taichung, Taiwan

**Keywords:** factor VII deficiency, FVII:c 681+1 G>T, IVS6+1G>T, cephalohematoma, intracranial hemorrhage, factor VII replacement therapy, neonatal coagulopathy

## Abstract

**Introduction:** Factor VII deficiency is a rare inherited autosomal recessive bleeding disorder with a global prevalence of 1/500,000. Most cases remain asymptomatic, and cases with severe clinical presentation are rarely reported.

**Case Presentation:** A newborn male with no relevant maternal antenatal history, delivered *via* vacuum-assisted cesarean section, presented with a large cephalohematoma after delivery. Poor appetite, pale appearance, and bulging fontanelles were observed 2 days later, progressing to hypovolemic shock. Further imaging examination revealed a large intracranial hemorrhage. Serial laboratory examination revealed remarkable coagulopathy with prolonged prothrombin time and factor VII deficiency (<1%, severe type). The patient was genetically confirmed to have the FVII:c 681+1 G>T homozygous mutation. Brain hemorrhage was resolved with high-dose factor VII replacement therapy with recombinant activated factor VII. However, repeated hemothorax and intracranial hemorrhage were detected. Therefore, the patient was under regular factor VII supplementation with a rehabilitation program for cerebral palsy.

**Conclusions:** A case of factor VII deficiency with large cephalohematoma and intracranial hemorrhage after birth is described herein, which was treated with high-dose replacement therapy. Variants of the FVII:c 681+1 G>T (IVS6+1G>T) homozygous genotype may present with a severe phenotype at the neonatal stage. We aim to share a unique neonatal presentation with a certain genotype and treatment experience with initial replacement therapy, followed by regular prophylactic dosage.

## Introduction

Factor VII (FVII) is a vitamin K-dependent serine protease that is activated by exposure to integral membrane protein tissue factor on the vascular lumen and triggers the initiation of blood clotting (1). Inherited factor VII deficiency is the most common among the rare congenital coagulation disorders and is characterized by autosomal recessive inheritance (2). However, inherited factor VII deficiency is usually diagnosed by laboratory workup after a bleeding episode. The global prevalence is 1/500,000 (2), while one-third of patients with factor VII deficiency tend to remain asymptomatic during their life (3). The prevalence of factor VII deficiency may be underestimated because of asymptomatic patients, even if their FVII:C ratio is decreased (2). The median age for the discovery of an inherited defect is 8 years (2). The symptoms also vary case by case. Most often, epistaxis (60%), gum bleeding (34%), easy bruising (36%), and menorrhagia have been reported. Males and females are equally affected; however, females are more likely to have symptomatic disease due to the symptoms of menorrhagia (3). Among these, only 10%-15% exhibit potentially life-threatening or limb-threatening hemorrhages (CNS, GI, or hemarthrosis). Severe bleeding usually occurs soon after birth, and CNS bleeding is even less frequent (2.5%); further cases of successful treatment in neonates with severe intraventricular hemorrhage are rarely reported (1, 3). In addition, there is no method to detect the severity, even though the FVII gene has already been sequenced (2). Herein, we present a 2-day-old male infant who experienced severe CNS bleeding at a very early stage with a lethal mutation in FVII:c 681+1 G>T.

## Case Presentation

**Figure 1:**
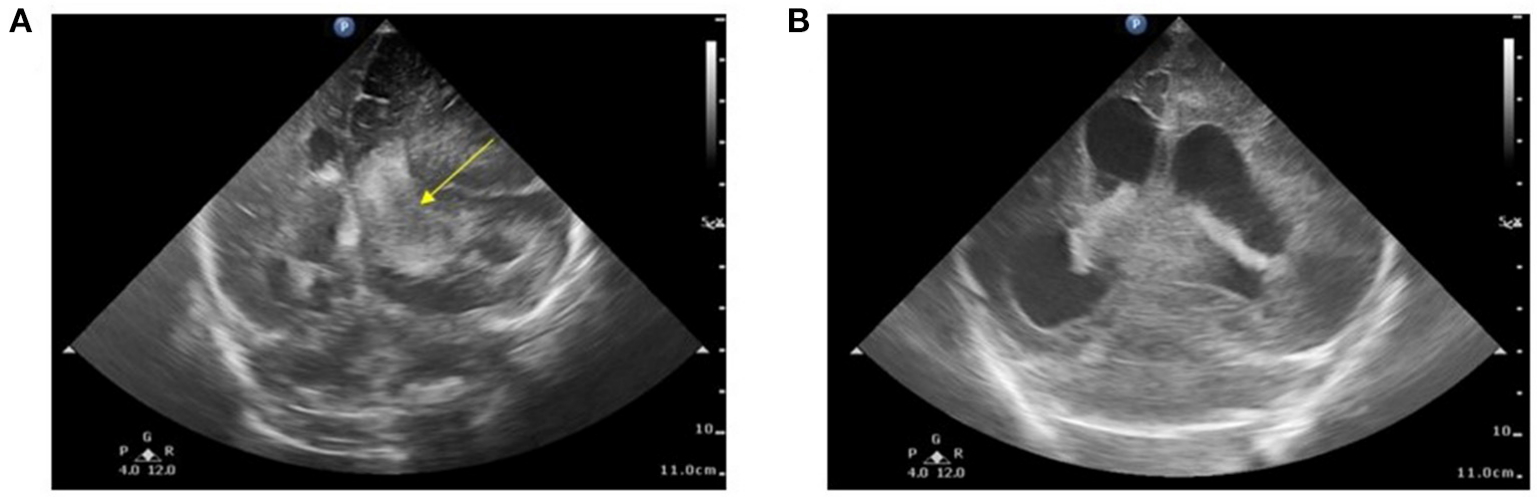
**(A)** Brain echo at 3 days of age. Bilateral intraventricular and intracranial hemorrhage with ventricular enlargement and midline shift to the right. The arrow indicates the blood clot involved in the left ventricle (Grade 3). **(B)** Brain echo at 16 days of age. Hemorrhage resolved after the 14-day replacement therapy. Image shows post-hemorrhagic hydrocephalus.

A 2-day-old male infant was born to a non-consanguineous asymptomatic couple at 36 weeks and 3 days of gestation. The mother reported no abnormal findings during antenatal examination and no relevant family history. Delivery was performed *via* cesarean section due to labor signs with breech presentation. A vacuum device was used during the delivery. After delivery, the infant had a large hematoma on the right side and was under routine care in the baby room. Brain echo performed on day of life (DOL) 1 reported left subependymal cysts with no evidence of hemorrhage. However, on DOL3, the infant began to show loss of appetite with coffee-ground vomitus. Physical examination revealed a pale appearance, similar cephalohematoma size compared to that at birth, and tachycardia. Upon transfer to a level III Medical Center on the same day, hypovolemic shock with tachycardia, hypotension, and bleeding tendency presenting as nasal and peripheral intravenous site bleeding were recorded. Brain echoes were arranged due to bulging fontanelles which revealed a right-sided massive intracranial hemorrhage (ICH) with a midline shift (Figure 1A). A subsequent CT scan revealed a large hemorrhage in the left frontal–temporal–parietal lobe with a diagnosis of intraventricular hemorrhage and post-hemorrhagic hydrocephalus.

**Figure 2:**
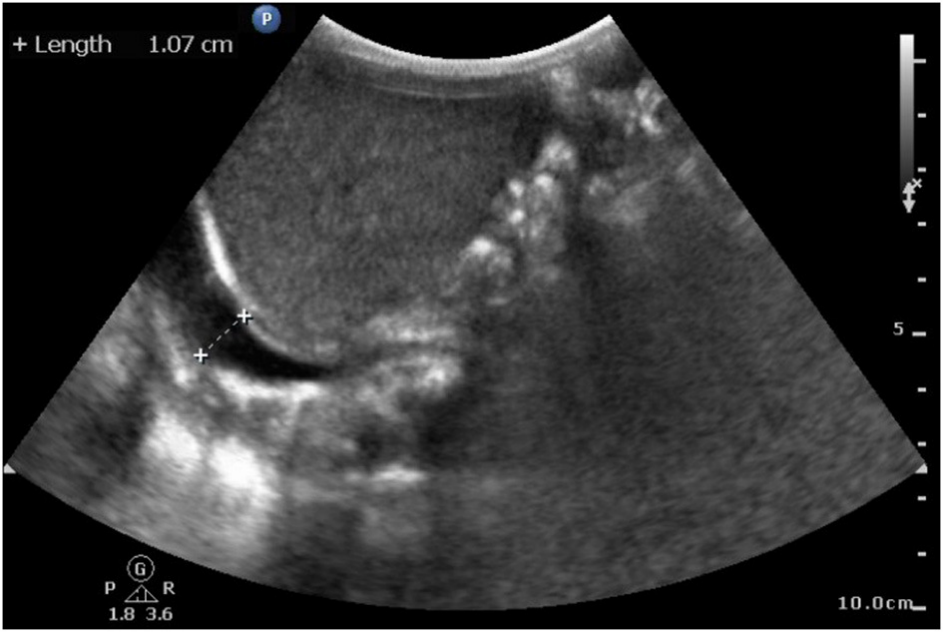
Sonography at 43 days of age. Hemothorax detected by chest sonography after first discharge without prophylaxis. Image shows the accumulation of clear fluid in the right pleural cavity under infra-hepatic view.

Initial blood tests revealed anemia (hemoglobin, 7.8 g/dl) and remarkable coagulopathy with prolonged prothrombin time (PT, 53.4 s with reagent RecombiPlasTin2G; aPTT, 44.5 s with reagent SynthASil). The baby was massively transfused with packed RBC and fresh frozen plasma, and vitamin K was administered repeatedly, but the coagulopathy persisted. A retractable prolonged PT was observed. Coagulopathy examination revealed factor VII activity level <1.0%, which led to a diagnosis of factor VII deficiency. Under clinical diagnosis, we surveyed the F7 exon region and exon ±5 bp and further confirmed the variant, FVII:c 681+1 G>T (IVS6+1G>T), by Sanger sequencing before they were clinically reported. Genome analysis of his parents and older sister revealed that they were heterozygous for the same mutation. Due to severe ICH, treatment with recombinant activated factor VII was provided with high-dose replacement therapy (30 μg/kg/dose Q3H) until hemorrhage resolved on sonography, which took 2 weeks (Figure 1B). The dosage was then increased to 90 μg/kg/dose Q3H for blood clot evacuation and VP shunt insertion. The baby was discharged at 35 days of age without prophylaxis with recombinant activated factor VII, considering the relative immobilization status during the neonatal stage. However, the patient presented with irritable crying and pale appearance 1 week after the first discharge after burping the baby. Progressive respiratory distress was observed. Hemothorax was detected by chest sonography (Figure 2) and diagnostic tapping, followed by treatment with high-dose recombinant activated factor VII (30 μg/kg/dose Q4H). There was a gradual improvement in clinical condition. Repeat chest radiography and ultrasonography revealed a reduction in the pleuritic fluid. Hence, port-A was administered and recombinant activated factor VII dosage was gradually weaned to a prophylactic dose of 30 μg/kg/dose twice a day. The patient was sustained for 1 month with port-A recombinant activated factor VII injection; however, he further encountered port-A infection (2.5 months old) with *Staphylococcus aureus*, and we removed the port-A catheter. The second intracranial hemorrhage occurred at 6 months of age, which was a week after discontinuation of prophylaxis therapy after port-A removal. Considering medical accessibility, we shifted to prothrombin complex concentrate (Beriplex) treatment because of its longer half-life. The patient is now undergoing a rehabilitation program with prothrombin complex concentrate prophylaxis through a port-a-catheter every 2 days without any thrombotic complications.

In our case, considering life-threatening status, we used recombinant activated factor VII (NovoSeven) at a dose of 30 μg/kg/dose every 3 h for 14 days, which successfully stopped the bleeding, and a 90-μg/kg/dose every 3 h for 7 days before major surgery (Table 1), which was effectively tolerable for the surgery. Further long-term prophylaxis with plasma-derived factor VII at the dosage of 35 IU/kg/dose every 2 days showed no bleeding symptoms observed except discontinuation of prophylaxis periods, which presented with hemothorax and ICH, respectively.

**Table 1:** Treatments and investigation results during the hospital course.

**Age**		**Dose**	**Daily dose**	**Treatment days**	**Events**
2-day	Replacement therapy	Recombinant activated Factor VII 30 μg/kg/dose Q3H	240 μg/kg/day	14	NA
16-day	Surgery	Recombinant activated Factor VII 90 μg/kg/dose Q3H	720 μg/kg/day	7	NA
23-day	Prophylaxis	Recombinant activated Factor VII 30 μg/kg/dose QOD	15 μg/kg/day	13	NA
36-day	No prophylaxis due to no central line			7	Hemothorax
43-day	Replacement therapy	Recombinant activated Factor VII 30 μg/kg/dose Q4H	180 μg/kg/day	21	NA
64-day	Prophylaxis	Recombinant activated Factor VII 30 μg/kg/dose BID	60 μg/kg/day	97	NA
161-day	No prophylaxis due to no central line			10	ICH
171-day	Prophylaxis	Plasma-derived Factor VII 35 IU/kg/dose QOD	17.5 IU/kg/day	Till now	NA

## Discussion

Factor VII has been fully sequenced since 1978 and could detect 90–92% of FVII mutations by sequencing (2). In our case, we used next-generation sequencing, which uses array-based sequencing to process reactions in parallel and enables the simultaneous investigation of multiple genes at a manageable cost, to identify the variants. While there is still little research on whether there is a relationship between different gene mutations and clinical presentations (2), nine cases of factor VII deficiency with mutation in FVII:c 681+1 G>T (IVS6+1G>T), as in our case, were identified in the literature review (Table 2) (4–11). Among them, six cases presented with severe clinical presentation, such as CNS bleeding or GI bleeding, according to the classification proposed by Mariani et al. in 2005 (12). Among the severe cases, 83% (5/6) were identified as homozygous genotypes. Only 16% (1/6) were heterozygous.

**Table 2:** Cases with FVII:c 681+1 G>T (IVS6+1G>T) mutation collected in literature.

**Case numbers**	**Zygosity**	**Onset age**	**Severity**	**References**
2	Homozygous	NB stage	Severe	(4)
1	Homozygous	NB stage	Severe	(5)
1	Heterozygous	NA	Mild	(6)
2	Homozygous	1-month-old and 4-month-old	Severe	(7)
1	Heterozygous	10-year-old	Severe	(8)
1	Heterozygous	NA	Mild	(9)
1	Heterozygous	NA	Mild	(10)

*NB, newborn, defined as age below 1 month of age; NA, not reported in the reference article*.

Inherited factor VII deficiency has a wide range of clinical manifestations; while laboratory workup was performed in the case of bleeding events, screening in the context of family history of factor VII deficiency may be a powerful predictor (2, 3). An isolated prolonged PT with normal aPTT strongly suggests the diagnosis of factor VII deficiency, which is characterized by an FVII:c below 70% (2). The reagents used for PT and factor VII activity measurements vary the factor VII deficiency sensitivity (2). Although factor VII activity may be easily measurable, bleeding tendency differs from case to case and may not be simply defined by factor VII activity alone (2). The activation of a cryptic donor splice site that induces the synthesis of an in-frame alternative FVII transcript can be responsible for the residual activity of F7, and this can possibly explain the clinical diversity (7). Fortunately, spontaneous major bleeding mostly occurs in severe cases where FVII activity is <8%; on the other hand, FVII levels > 20% can protect from spontaneous bleeding in most cases (2, 3). Our case initially presented with cephalohematoma, which is a common birth injury. However, among neonates born with cephalohematoma, a study revealed that up to 36.8% had ICH (13). In neonates with hemophilia, the incidence of cranial bleeds (intra- and extracranial) present at birth accounts for ~3.5% within the first 28 days (14). The incidence rate of ICH in newborns is affected by timing as well as the sensitivity of the imaging modality (brain ultrasound vs. brain MRI) (15). The prevalence of asymptomatic ICH may have been underestimated. Although most cephalohematoma will spontaneously resolve without complications, hemorrhagic disorder should be considered when it is accompanied by other bleeding symptoms.

Spontaneous hemothorax occurring within a week in our case showed a strong relationship with factor VII deficiency. Spontaneous hemothorax is a sparse report in children, even in hemophilia (16). Consequently, there are few reports on management of this rare presentation, and few successful reports have declared that conservative management works (16, 17). We opted to drain the effusion because of respiratory distress, a relatively life-threatening condition, and successful treatment combined with high-dose recombinant activated factor VII at a dose of 30 μg/kg/dose every 4 h. This subsequent episode reinforces the necessity of prophylaxis in a severe phenotype. The most frequent indications for long-term prophylaxis were CNS bleeding (58%), hemarthrosis (15%), and GI bleeding (9%), while the prophylaxis dosages ranged (18). Long-term prophylaxis should be considered and started soon after the first clinically significant bleeding in severe forms of FVII deficiency.

Guidelines for the management of this rare bleeding disorder are lacking. Factor VII level alone cannot drive management because it cannot predict bleeding risk tendency (3). Replacement therapy is the main therapeutic option for inherited factor VII deficiency in severe cases. Replacement options are characterized by peculiar features. Recombinant activated factor VII stands out due to the advantage of efficacy and has no risk of transmitting pathogens, while the disadvantages might be the higher cost per dose (1, 19). Plasma-derived factor VII is also an effective choice at a lower price, while other vitamin K-dependent factor concentrations will result in higher levels of prothrombin and may be associated with thrombosis, which is still a suitable option for replacement therapy (19). The dosage and schedules for different situations (spontaneous bleeding episodes, major surgery, invasive procedures, and prophylaxis) have been reported in multicenter observational studies (3). According to recent reports, the recommended dose per injection is 15–30 μg/kg every 4–6 h on demand, and long-term prophylaxis is highly recommended in severe cases based on the patient's clinical and family history (20). The treatment for major surgery is recommended at a dose of 15–30 mg/kg before surgery every 4–6 h, in the first 24 h, then increasing the interval to 8–12 h (20). In patients with factor 7 deficiency undergoing surgery and during bleeding episodes, a continuous infusion of recombinant activated factor VII has also been reported with regard to hemostatic efficacy, safety, and cost (21, 22).

In our case, parental and sibling heterozygous carriers of the same point were reported, and there was no family consanguinity. This mutation identified in our patient is homozygous, which may present with a more severe phenotype based on current evidence. This information allowed us to exclude a compound heterozygous deficiency state in a subsequent pregnancy using PCR/direct sequencing of the FVII gene.

In conclusion, we report a case of severe FVII deficiency with a high-risk lethal FVII:c 681+1 G>T homozygous mutation encountered due to ICH at a very early stage. High-dose replacement therapy followed by blood clot evacuation surgery is required. Clinicians should be aware of hemorrhagic episodes in newborns for the purpose of early diagnosis of possible congenital bleeding disorders to provide the earliest appropriate treatment.

## Data Availability Statement

The original contributions presented in the study are included in the article/supplementary material, further inquiries can be directed to the corresponding author.

## Ethics Statement

Written informed consent was obtained from the minor(s)' legal guardian/next of kin for the publication of any potentially identifiable images or data included in this article.

## Author Contributions

Y-CL contributed to writing the manuscript. Y-TC and C-TP were responsible for patient care and manuscript preparation. All authors read and approved the final manuscript.

## Funding

This study was supported in part by the China Medical University Hospital (Grant Number: DMR-109-177).

## Conflict of Interest

The authors declare that the research was conducted in the absence of any commercial or financial relationships that could be construed as a potential conflict of interest.

## Publisher's Note

All claims expressed in this article are solely those of the authors and do not necessarily represent those of their affiliated organizations, or those of the publisher, the editors and the reviewers. Any product that may be evaluated in this article, or claim that may be made by its manufacturer, is not guaranteed or endorsed by the publisher.
